# The Objective Physical Activity and Cardiovascular Disease Health in Older Women (OPACH) Study

**DOI:** 10.1186/s12889-017-4065-6

**Published:** 2017-02-14

**Authors:** Andrea Z. LaCroix, Eileen Rillamas-Sun, David Buchner, Kelly R. Evenson, Chongzhi Di, I-Min Lee, Steve Marshall, Michael J. LaMonte, Julie Hunt, Lesley Fels Tinker, Marcia Stefanick, Cora E. Lewis, John Bellettiere, Amy H. Herring

**Affiliations:** 1 Division of Epidemiology, Department of Family Medicine and Public Health, University of California San Diego, La Jolla, CA USA; 2 Division of Public Health Sciences, Fred Hutchinson Cancer Research Center, Seattle, WA USA; 3 University of Illinois at Urbana-Champaign, Champaign, IL USA; 4 Department of Epidemiology, Gillings School of Global Public Health, University of North Carolina Chapel Hill, Chapel Hill, NC USA; 5 Division of Preventive Medicine, Brigham and Women’s Hospital, Harvard Medical School, Boston, MA USA; 6 Department of Epidemiology and Environmental Health, School of Public Health and Health Professions, University at Buffalo - SUNY, Buffalo, NY USA; 7 Stanford Prevention Research Center, Stanford University School of Medicine, Stanford University, Stanford, CA USA; 8 Department of Medicine, Division of Preventive Medicine, University of Alabama Birmingham, Birmingham, AL USA

**Keywords:** Physical activity, Sedentary behavior, Older women, Postmenopausal, Accelerometer, Sleep, Cardiovascular disease, Falls, Mortality

## Abstract

**Background:**

Limited evidence exists to inform physical activity (PA) and sedentary behavior guidelines for older people, especially women. Rigorous evidence on the amounts, intensities, and movement patterns associated with better health in later life is needed.

**Methods/Design:**

The Objective PA and Cardiovascular Health (OPACH) Study is an ancillary study to the Women’s Health Initiative (WHI) Program that examines associations of accelerometer-assessed PA and sedentary behavior with cardiovascular and fall events. Between 2012 and 2014, 7048 women aged 63–99 were provided with an ActiGraph GT3X+ (Pensacola, Florida) triaxial accelerometer, a sleep log, and an OPACH PA Questionnaire; 6489 have accelerometer data. Most women were in their 70s (40%) or 80s (46%), while approximately 10% were in their 60s and 4% were age 90 years or older. Non-Hispanic Black or Hispanic/Latina women comprise half of the cohort. Follow-up includes 1-year of falls surveillance with monthly calendars and telephone interviews of fallers, and annual follow-up for outcomes with adjudication of incident cardiovascular disease (CVD) events through 2020. Over 63,600 months of calendar pages were returned by 5,776 women, who reported 5,980 falls. Telephone interviews were completed for 1,492 women to ascertain the circumstances, injuries and medical care associated with falling. The dataset contains extensive information on phenotypes related to healthy aging, including inflammatory and CVD biomarkers, breast and colon cancer, hip and other fractures, diabetes, and physical disability.

**Discussion:**

This paper describes the study design, methods, and baseline data for a diverse cohort of postmenopausal women who wore accelerometers under free-living conditions as part of the OPACH Study. By using accelerometers to collect more precise and complete data on PA and sedentary behavior in a large cohort of older women, this study will contribute crucial new evidence about how much, how vigorous, and what patterns of PA are necessary to maintain optimal cardiovascular health and to avoid falls in later life.

**Clinical trials registration:**

ClinicalTrials.gov identifier NCT00000611. Registered 27 October 1999.

**Electronic supplementary material:**

The online version of this article (doi:10.1186/s12889-017-4065-6) contains supplementary material, which is available to authorized users.

## Background

Due to their longevity advantage, women outnumber men in later life [1]. By the year 2060, the number of women ages 65 years and older in the United States (US) is estimated to be 52.7 million [2]. Despite their greater longevity, women have more morbidity and disability than men [3], resulting in higher frequency of outpatient visits and hospitalizations, use of long-term care services, and health care expenditures [4–6].

Regular physical activity (PA) provides remarkable health benefits for older adults, including reducing the risk of mortality, developing many chronic diseases, functional limitations, and fall-related injuries [7]. The 2008 PA Guidelines for Americans recommends that adults of all ages get a minimum of 150 min per week of moderate-intensity aerobic activity [7], however, accelerometer-measured PA data estimated that only 2% of older Americans met this guideline [8] and they sat up to 11 h per day [9–11]. Women, especially older women, were underrepresented in the evidence reviewed to derive these guidelines. This paper describes the study design, methods, and baseline data for a diverse cohort of postmenopausal women in the “Objective PA and Cardiovascular Health in Older Women” (OPACH) Study [R01 HL105065; PI: A LaCroix]. This study will contribute crucial new evidence about how much, how vigorous, and what patterns of PA are necessary to maintain optimal cardiovascular health in later life and whether PA levels are associated with incident falls.

## Methods

### Study population

#### The Women’s Health Initiative, Extension Studies and Long Life Study

The OPACH Study is an ancillary study to the WHI study, a major National Institutes of Health (NIH) research program that began in the early 1990s. Postmenopausal women ages 50 to 79 years were enrolled in the WHI Clinical Trials or the Observational Study from 40 clinical sites throughout the US from 1993 to 1998. Details about WHI have been extensively described [12, 13]. Protocols were approved by institutional review boards at participating institutions and all women gave written informed consent. WHI participants continue to be followed annually for disease events, changes in functional status, and death through the main program that ended in 2005, and three Extension Studies (2005–2010; 2010–2015; 2015–2020). Enrollment in the Extension Studies required that eligible women (alive and willing to be contacted) provide informed consent for continued follow-up in WHI. In the first Extension, 76.9% of 150,075 eligible women consented to further follow-up. In the second Extension, 86.8% of 107,706 eligible women consented to further follow-up without a defined end date.

The WHI Long Life Study was conducted during the second WHI Extension Study among a subcohort of 7,875 WHI participants from March 2012 to May 2013 from all 40 original US clinical centers in their homes to collect new data to support research on factors associated with healthy aging and changing levels of biomarkers of CVD risk. Data collection included a brief clinical assessment (height, weight, waist circumference, blood pressure, and pulse), assessment of functional status (Short Physical Performance Battery (SPPB), [14, 15] and grip strength), and phlebotomy.

A total of 7,048 WHI women who consented to participate in both the Long Life Study and the OPACH study were provided with an ActiGraph GT3X+ (Pensacola, Florida) triaxial accelerometer, a sleep log, and an OPACH PA Questionnaire (Additional file 1) between March 2012 and April 2014 either during the home visit or via express mail afterwards.

### Cardiovascular disease outcomes

The primary outcomes for the OPACH Study are total cardiovascular disease (CVD) events and total mortality. WHI Extension Study participants are mailed forms annually to ascertain updates to their medical history. Medical records are obtained for reported outcomes and adjudicated by trained study physicians. Definitions of CVD outcomes are described in detail elsewhere [16] and summarized in Table 1. Deaths are ascertained when a family member informs the WHI staff, and through National Death Index searches and obituary notices. Participants are followed until they die, are lost-to-follow-up, or request no further contact. Vital status was known for 98% of women as of the end of 2014. Causes of death are determined based on available medical records, autopsy reports, and the death certificate in a blinded fashion by local and central physician adjudicators.

**Table 1:** Cardiovascular Disease and Mortality Outcomes Ascertainment in WHI

Study Outcome	Definition and Confirmation of Outcomes
Non-fatal myocardial infarction	Standardized criteria for diagnostic electrocardiography changes, elevated cardiac enzymes or both
Revascularization	Documentation of the procedure in the medical record.
Angina	Hospital record, angiography evidence, diagnostic stress test, or documented physician diagnosis and medical treatment
Congestive heart failure	Hospital record and diagnostic confirmatory tests
Fatal coronary heart disease	Documentation in the hospital record, autopsy report or cause of death on the death certificate with evidence of previous coronary heart disease
Stroke	Documentation in the medical record of neurologic deficit of rapid onset consistent with stroke and lasting for at least 24 h or until death
Death from other cardiovascular disease	Confirmatory evidence in the medical records as ascertained by physician adjudicators

### OPACH falls surveillance and data collection

To monitor safety, the OPACH study conducted surveillance on incident falls for one year after accelerometry and collected information about all injuries and fall-related injuries that required medical care. Incident falls were ascertained using a 13-month calendar distributed to OPACH participants to record daily if they had a fall (“no fall” or “yes, I fell”). Calendar pages were sent back monthly, data were entered and dates of reported falls were tracked. Participants were mailed a reminder postcard if calendars were not returned and, if a woman lost or misplaced any calendar pages, a new calendar was sent. The first month of each woman’s calendar corresponded to the month that she wore the accelerometer. Falls surveillance began in March 2012 and was completed in March 2015.

When a fall was reported on a calendar page, participants were interviewed by telephone about the circumstances of the fall, including events leading up to the fall, physical activities (both leisure and non-leisure) engaged in at the time of the fall, location of the fall (inside or outside of the home), whether injuries resulted and, if so, the body region and type of injury (particularly, any fractures), and the highest level of medical care received for all injuries. For women who fell multiple times within a month, data collection was limited to the first two injury falls and first two non-injury falls. Participants could be contacted multiple times if they reported falling on more than one calendar page over the 13-month follow-up period. Due to limited resources, beginning in April 2013, interviews were conducted among 100% of falls reported by the most physically active women - defined as those in the highest quintile (≥21 MET-hours/week) of self-reported total recreational PA - and 20% of falls reported from all other women were selected to be interviewed [17].

**Fig. 1:**
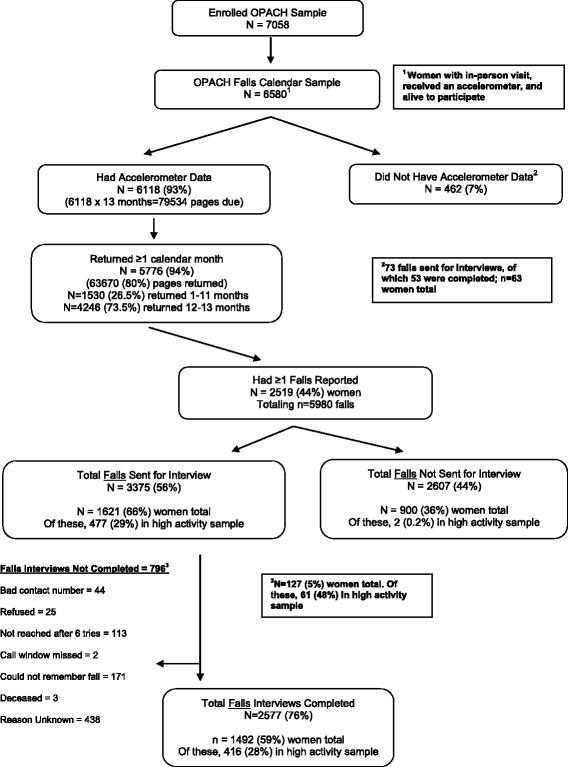
Strobe Diagram for Falls Surveillance in the OPACH Study

As shown in Fig. 1, 6,580 women received a 13-month calendar for falls surveillance, of whom, 6,118 (93%) returned an accelerometer with usable data. Of these, 5,776 (94%) women returned at least 1 month of calendar pages and 4,246 (69%) women returned 12–13 months of calendar pages. Among the over 63,600 pages of calendar months returned, 5,980 falls were reported and 3,375 of these falls were sent for interviews. A total of 2,577 (76%) fall interviews were completed from 1,492 women, 416 (28%) of whom were among the most physically active. Reasons that fall interviews were not completed included that the participant did not remember falling, could not be contacted, refused the interview, or was deceased.

### Accelerometer data collection

**Fig. 2:**
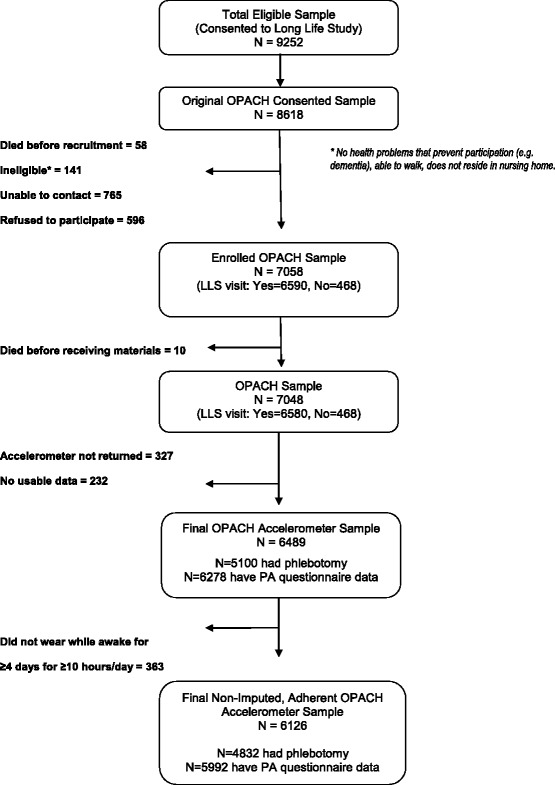
Strobe Diagram for Accelerometer Data in the OPACH Study

The hip-worn accelerometer was placed at the iliac crest and secured with a belt. Women were asked to wear the accelerometer for 7 days during both waking and sleeping hours, except when bathing or swimming, starting the day after they complete their home visit or received their mailed package. To isolate sleeping time, participants recorded time in and out of bed on the OPACH sleep log [18]. The accelerometer was preset to begin data collection at a specified date and time. The device provided no feedback to the participant about their PA. As shown in Fig. 2, 6,721 (95%) women returned their accelerometers and of these, 6,489 (92%) had some data for analysis. The 569 women who did not return accelerometers with usable data were somewhat more likely to be African-American, had poorer self-reported health, lower physical function scores, higher levels of depressive symptoms and higher prevalence of past cardiovascular disease, but were less likely to report a fall in the past year when compared to the women with accelerometer data.

### Accelerometer data processing

Accelerometer data were measured and saved at a rate of 30 times per second (i.e., at 30 hertz). When devices were returned from participants, the data were downloaded and saved for long-term storage. Over the data collection phase of the study, the ActiGraph software (ActiLife) versions 6.0.0 to 6.101 were used. Data were processed using ActiLife Firmware v2.4 and the activity counts for the three orthogonal axes at which acceleration was measured were output for every 15-s epoch using the normal frequency filter mode and the low frequency filter mode, separately. Data from the three axes were used to compute the vector magnitude (VM) by taking the square root of the sum of the vertical axis squared, the anterior-posterior axis squared, and the medial-lateral axis squared.

A computer-based automated algorithm, in alignment with the sleep logs and visual inspection, was used to identify the window of days with the maximum amount wear over a consecutive 7 day period [18]. When available, the sleep log data were used to identify periods when the participant reported being out of bed (vs. in bed). To maximize the use of accelerometer data when sleep logs were missing or were suspected to have reporting error, mean values for in-bed and out-of-bed times were imputed for each participant if at least one day of sleep log data were recorded. Population mean in-bed and out-of-bed times were used for women with no sleep log data. Automated methods for identifying in-bed periods are currently being explored [19, 20].

Accelerometer non-wear was defined by an interval of at least 90 consecutive minutes of zero VM counts per minute, with allowance for 2-min windows including nonzero VM counts as long as no counts were detected during the 30 min upstream and downstream of each window and that the cumulative duration of consecutive upstream and downstream zeros were ≥90 min [21, 22]. Any nonzero VM counts (except the allowed short intervals) were considered wear time.

An adherent day was defined as ≥10 h of accelerometer wear time during periods the participant was out-of-bed.

### Covariate data collection

The OPACH PA Questionnaire [see Additional File 1] was completed by women in their homes and mailed back to the WHI Clinical Coordinating Center for data entry. Table 2 (below) summarizes the measures included in the questionnaire and the origin of validated scales.

**Table 2:** Summary and sources of measures used in OPACH Physical Activity Questionnaire

Survey Measure	Question Number	Source (within References)
WHI self-reported physical activity	1–4	[17]
Borg scale assessment of relative intensity	5	[32]
Rating of perceived capacity scale	6–7	[33]
WHI self-reported sedentary behavior	8–10	[17]
CARDIA study sedentary behavior scale	11–12	[34, 35]
Short Falls Efficacy Scale International	13	[36]
Participation in falls prevention programs	18–19	Original, newly developed questions
Falls history assessment	20–21	[37]
Engagement in physical activity during fall and injurious fall; injurious fall history assessment	22–26	Original, newly developed questions
Urban environment and physical activity	27	[38]
Neighborhood Environmental Walkability Scale - select subscales (neighborhood type and crime safety)	28–29	[39]
CHAMPS self-reported physical activity assessment	30	[40]

*Abbreviations*: *CARDIA* Coronary Artery Risk Development in Young Adults, *CHAMPS* Community Health Activities Model Program for Seniors, *WHI* Women’s Health Initiative

The health status of OPACH participants has been extensively and continuously characterized since their WHI enrollment in 1993–1998. Information was collected by interview and/or self-administered questionnaires for all participants on age, race/ethnicity, education, age at menopause, hormone therapy use, medication use (e.g., statins, lipid-lowering drugs, antihypertensive drugs), treated diabetes, personal and parental history of major chronic diseases, and physical functioning using the RAND SF-36 instrument [23]. A medication inventory was collected by mail just prior to the start of the Long Life Study.

As part of the Long Life Study protocol, a fasting blood draw was collected during the participant’s home visit. Biomarkers (glucose, insulin, creatinine, high-sensitivity C-reactive protein, high and low density lipoprotein cholesterol, triglyceride, total cholesterol) were measured at the University of Minnesota. Of the 7,875 Long Life Study participants, 7,325 (93%) have biomarker data available. Of these, 5,100 (70%) participated in the OPACH study.

### OPACH calibration study

No standard was available to classify intensity of PA, and to distinguish sedentary behavior from light PA, using accelerometer data in older adults. Therefore, we conducted a separate laboratory-based calibration study in women ages 60 to 91 years to determine accelerometer count cutpoints that best distinguish levels of PA volume intensity in older women. Details of the calibration study design and results have been published previously [24].

**Fig. 3:**
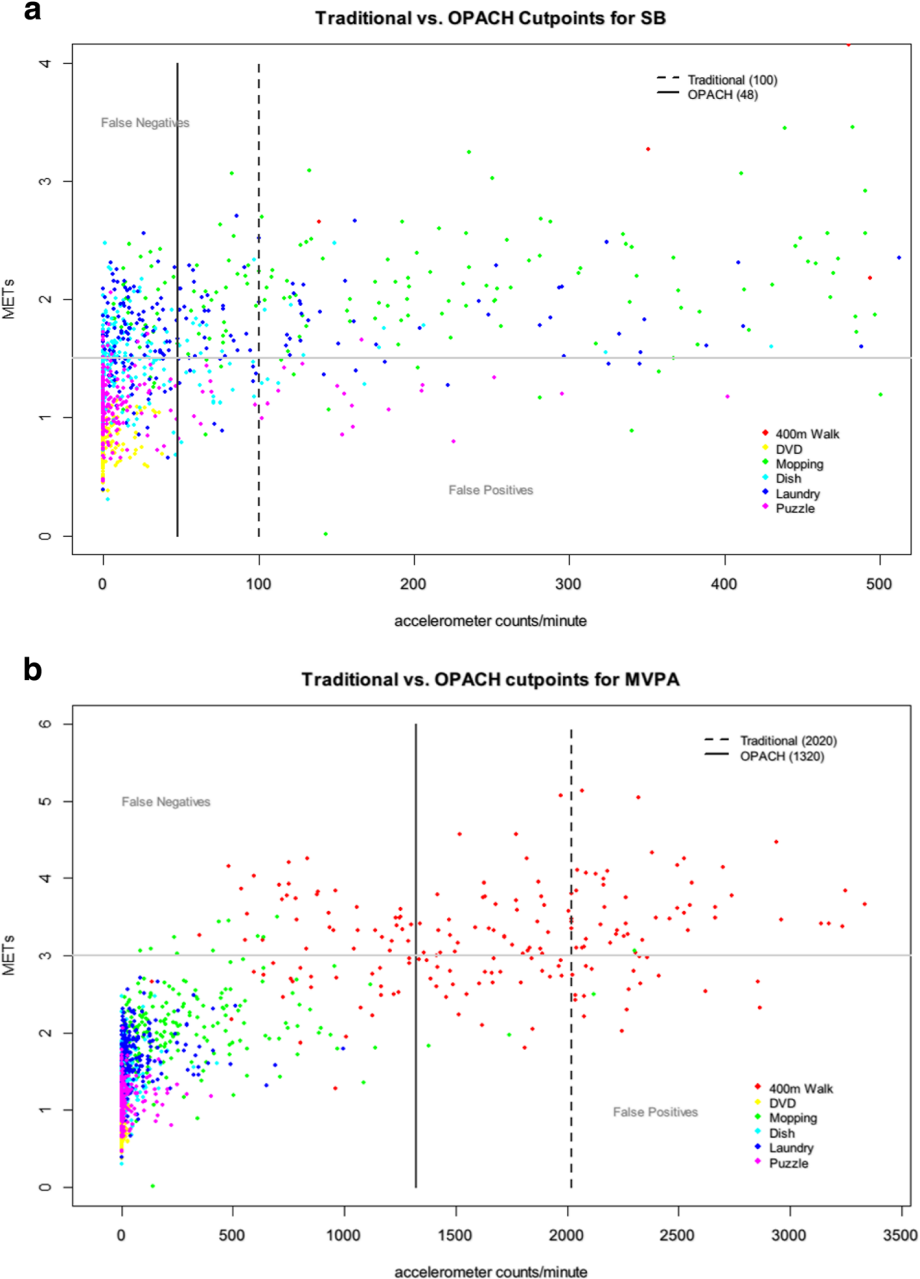
**a** Traditional vs. OPACH Calibration Study Cutpoints for Sedentary Behavior. **b** Traditional vs. OPACH Calibration Study Cutpoints for Moderate-to-Vigorous Intensity Physical Activity

Traditional accelerometer cutpoints were found to be too high for older women resulting in PA being underestimated [11, 25]. Traditional cutpoints were derived using vertical axis accelerometer counts, so Fig. 3a and b present OPACH vertical axis count cutpoints to allow for accurate comparisons. Figure 3a displays two different vertical axis count cutpoints for light intensity PA in OPACH calibration study participants. The traditional cutpoint for sedentary behavior of <100 counts per minute on the vertical axis [10] results in more light intensity PA minutes being misclassified as sedentary (false negatives) causing an underestimation of light intensity PA minutes and overestimation of sedentary minutes among OPACH participants. Similarly, Fig. 3b shows that the vertical axis count cutpoint for moderate-to-vigorous PA (MVPA) from the calibration study (> = 1320 counts/minute) was lower than a commonly used cutpoint (> = 1952 counts/minute) [26]. Again, using the higher cutpoint would cause an underestimation of MVPA for women in the OPACH study.

The OPACH Calibration Study reported intensity cutpoints derived from different methods of analyzing the data [24]. OPACH chose the method that defined a MET as 3.0 ml/kg/min and with data processed using the normal frequency filter, where cutpoints were derived by balancing false positive and false negatives. A MET value of 3.0 ml/kg/min is slightly lower than the traditionally defined value of 1 MET = 3.5 ml/kg/min, but better reflects observed resting energy expenditure of older adults studied with indirect calorimetry [24, 27, 28]. This resulted in the categorization of PA intensity as shown in Table 3.

**Table 3:** Categorization of Physical Activity Intensity Levels for Older Women in the OPACH Calibration Study

Intensity Level	Vector Magnitude Cutpoint Values (counts per 15-s)
Sedentary	0–18
Low light	19–225
High light	226–518
Moderate-to-Vigorous	≥519

### Baseline characteristics

Table 4 describes demographic, health behavior and health status characteristics by four age groups for the 6,489 women with accelerometer data. Most women were in their 70s (40%) or 80s (46%), while approximately 10% were in their 60s and 4% were age 90 years or older. Non-Hispanic Black and Hispanic/Latina women represented half of the sample and were generally younger than the Non-Hispanic White women in the study. Physical functioning, measured both through the RAND SF-36 survey [23] and the SPPB test [14], decreased as age increased. Likewise, self-reported PA levels decreased across incremental age groups. As women aged, they were more likely to have fallen in the past year and were more concerned about falling in the future. Average resting systolic blood pressure (SBP) was higher as age increased; however, average resting diastolic blood pressure, waist circumference, weight, and body mass index (BMI) were lower.

**Table 4:** Baseline characteristics of OPACH participants by age group

		Age Group, years				
	Total	63–69	70–79	80–89	90+	*p*-value
N (%)	6489	666 (10.3)	2600 (40.1)	2953 (45.5)	270 (4.2)	
Race/Ethnicity, n (%)						
Non-Hispanic White	3205 (49.4)	94 (2.9)	697 (21.8)	2192 (68.4)	222 (6.9)	<0.001
Non-Hispanic Black	2187 (33.7)	373 (17.1)	1264 (57.8)	511 (23.4)	39 (1.8)	
Hispanic/Latina	1097 (16.9)	199 (18.1)	639 (58.3)	250 (22.8)	9 (0.8)	
Education, n (%)						
High School or less	1316 (20.4)	101 (7.7)	499 (37.9)	652 (49.5)	64 (4.9)	0.002
Some College	2496 (38.7)	267 (10.7)	1024 (41.0)	1111 (44.5)	94 (3.8)	
College Graduate or more	2634 (40.9)	293 (11.1)	1056 (40.1)	1174 (44.6)	111 (4.2)	
Current Smoker, n (%)	164 (2.8)	35 (21.3)	81 (49.4)	47 (28.7)	1 (0.6)	<0.001
Alcohol Use, n (%)						
Non-drinker	2230 (37.9)	203 (9.1)	912 (40.9)	1022 (45.8)	93 (37.8)	0.12
< 1 drink per week	2008 (34.1)	210 (10.5)	820 (40.8)	889 (44.3)	89 (36.2)	
≥ 1 drink per week	1651 (28.0)	176 (10.7)	617 (37.4)	794 (48.1)	61 (26.0)	
Excellent or Very Good Self-Rated Health, n (%)	2977 (50.8)	338 (11.4)	1216 (40.9)	1321 (44.4)	102 (3.4)	<0.001
RAND SF-36 Physical Function Score, mean (SD)	67.9 (26.0)	78.9 (23.1)	73.0 (25.0)	62.5 (25.3)	48.3 (25.3)	<0.001
Depressive Symptoms Score, mean (SD)	0.03 (0.11)	0.04 (0.15)	0.03 (0.12)	0.02 (0.09)	0.01 (0.07)	<0.001
Self-Reported Physical Activity, MET-hrs/week, mean (SD)	11.9 (14.0)	15.0 (16.5)	13.3 (14.8)	10.4 (12.8)	6.6 (8.9)	<0.001
CHAMPS Expenditure, MET-hrs/week, mean (SD)	29.1 (26.8)	34.8 (32.2)	31.7 (29.0)	26.5 (23.4)	18.9 (18.3)	<0.001
CARDIA Scale, sedentary hours/week, mean (SD)	56.4 (22.7)	58.3 (25.3)	57.8 (24.1)	54.7 (20.6)	54.7 (20.9)	<0.001
Falls in past 12 months, n (%)						
None	4503 (69.4)	493 (11.0)	1870 (41.5)	1974 (43.8)	166 (3.7)	<0.001
One time	1258 (19.4)	112 (8.9)	484 (38.5)	602 (47.9)	60 (4.8)	
Two or more times	728 (11.2)	61 (8.4)	246 (33.8)	377 (51.8)	44 (6.0)	
Falls Efficacy Score, mean (SD)	10.7 (4.2)	9.6 (3.8)	10.1 (3.8)	11.3 (4.3)	13.3 (5.1)	<0.001
Short Physical Performance Battery score, mean (SD)	8.2 (2.5)	9.2 (2.1)	8.6 (2.3)	7.7 (2.6)	6.5 (2.9)	<0.001
Systolic Blood Pressure, mean (SD)	125.7 (14.3)	122.6 (12.9)	125.2 (13.8)	126.6 (14.7)	127.9 (16.4)	<0.001
Diastolic Blood Pressure, mean (SD)	72.5 (8.8)	73.6 (8.1)	73.3 (8.6)	71.8 (9.0)	71.0 (9.2)	<0.001
Waist, inches, mean (SD)	35.5 (5.5)	36.1 (5.5)	36.1 (5.7)	35.0 (5.2)	33.8 (5.1)	<0.001
Weight, lbs, mean (SD)	158.4 (34.4)	171.8 (37.5)	166.1 (35.9)	150.6 (30.0)	138.3 (25.9)	<0.001
Body Mass Index, kg/m^2^, mean (SD)	28.2 (5.8)	29.9 (6.2)	29.3 (6.1)	27.1 (5.2)	25.9 (4.8)	<0.001
Body Mass Index Categories, n (%)						
< 25 kg/m^2^	1944 (32.1)	128 (6.6)	627 (32.3)	1065 (54.8)	124 (6.4)	<0.001
25–<30 kg/m^2^	2199 (36.3)	209 (9.5)	868 (39.5)	1032 (46.9)	90 (4.1)	
≥ 30 kg/m^2^	1917 (31.6)	259 (13.5)	938 (48.9)	677 (35.3)	43 (2.2)	
Medical History						
Stroke	459 (7.1)	21 (4.6)	134 (29.2)	271 (59.0)	33 (7.2)	<0.001
Congestive Heart Failure	133 (2.1)	9 (6.8)	41 (30.8)	73 (54.9)	10 (7.5)	0.013
Total CVD	1327 (20.5)	92 (6.9)	472 (35.6)	687 (51.8)	76 (5.7)	<0.001
Hip fracture	188 (2.9)	4 (2.1)	24 (12.8)	133 (70.7)	27 (14.4)	<0.001
Any clinical fracture	2202 (33.9)	150 (6.8)	775 (35.2)	1156 (52.5)	121 (5.5)	<0.001
Diabetes	2078 (32.0)	237 (11.4)	933 (44.9)	846 (40.7)	62 (3.0)	<0.001
Osteoarthritis	4000 (61.6)	404 (10.1)	1599 (40.0)	1817 (45.4)	180 (4.5)	0.36
Invasive cancer	992 (15.3)	72 (7.3)	391 (39.4)	488 (49.2)	41 (4.1)	0.003
Breast cancer	480 (7.4)	44 (9.2)	216 (45.0)	202 (42.1)	18 (3.8)	0.15

*Abbreviations*: *CARDIA* Coronary Artery Risk Development in Young Adults, *CHAMPS* Community Health Activities Model Program for Seniors, *CVD* cardiovascular disease, *MET* Metabolic equivalents, *OPACH* Objective Physical Activity and Cardiovascular Health, *SD* Standard deviation

Of the 6,489 women with accelerometer data, 6,126 wore the accelerometer while out of bed for at least 4 days for at least 10 h each day. Table 5 shows the characteristics of adherent women by quartiles of average VM counts per 15-s epoch. Consistent with the self-reported PA levels in Table 4, younger women had higher levels of PA. White women were more likely (30%) to be in the lowest PA quartile compared with Black women (24%) and Hispanic/Latina women (13%). Women in the highest quartile of PA had a higher frequency of excellent or very good self-rated health (33 vs. 16% among women in the lowest quartile of PA) and of not falling in the past year. Furthermore, physical functioning and self-reported PA were higher and average SBP, waist circumference, weight, and BMI were lower among women in the highest quartile of PA.

**Table 5:** Baseline characteristics of 6,126 OPACH participants by quartiles of accelerometer measured physical activity

		Quartiles of average VM per 15-s epoch (adherent days only)				
	Total	<70.7	70.7–<95.2	95.2–<126.0	≥126.0	*p*-value
Age group, years, n (%)						
63–69	627 (10.2)	67 (10.7)	98 (15.6)	169 (27.0)	293 (46.7)	<0.001
70–79	2448 (40.0)	417 (17.0)	600 (24.5)	664 (27.1)	767 (31.3)	
80–89	2794 (45.6)	908 (32.5)	767 (27.5)	663 (23.7)	456 (16.3)	
90+	257 (4.2)	139 (54.1)	67 (26.1)	36 (14.0)	15 (5.8)	
Race/Ethnicity, n (%)						
Non-Hispanic White	3046 (49.7)	911 (29.9)	779 (25.6)	726 (23.8)	630 (20.7)	<0.001
Non-Hispanic Black	2047 (33.4)	489 (23.9)	541 (26.4)	519 (25.4)	498 (24.3)	
Hispanic/Latina	1033 (16.9)	131 (12.7)	212 (20.5)	287 (27.8)	403 (39.0)	
Education, n (%)						
High School or less	1237 (20.3)	311 (25.1)	315 (25.5)	307 (24.8)	304 (24.6)	0.05
Some College	2349 (38.6)	620 (26.4)	610 (26.0)	555 (23.6)	564 (24.0)	
College Graduate or more	2499 (41.1)	590 (23.6)	593 (23.7)	655 (26.2)	661 (26.5)	
Current Smoker, n (%)	159 (2.9)	58 (36.5)	38 (23.9)	38 (23.9)	25 (15.7)	0.001
Alcohol Use, n (%)						
Non-drinker	2089 (37.4)	589 (28.2)	571 (27.3)	500 (23.9)	429 (20.5)	<0.001
< 1 drink per week	1912 (34.2)	477 (25.0)	479 (25.1)	490 (25.6)	466 (24.4)	
≥ 1 drink per week	1589 (28.4)	290 (18.3)	342 (21.5)	429 (27.0)	528 (33.2)	
Excellent or Very Good Self-Rated Health, n (%)	2852 (51.3)	469 (16.4)	665 (23.3)	781 (27.4)	937 (32.9)	<0.001
RAND SF-36 Physical Function Score, mean (SD)	68.2 (25.8)	50.4 (26.5)	66.0 (24.7)	73.4 (22.0)	82.0 (18.6)	<0.001
Depressive Symptoms Score, mean (SD)	0.03 (0.11)	0.03 (0.12)	0.03 (0.10)	0.02 (0.10)	0.02 (0.10)	0.31
Self-Reported Physical Activity, MET-hrs/week, mean (SD)	12.0 (14.1)	6.3 (9.1)	9.9 (11.1)	13.3 (13.9)	18.4 (17.6)	<0.001
CHAMPS Expenditure, MET-hrs/week, mean (SD)	29.4 (26.9)	18.8 (18.1)	25.0 (20.7)	32.4 (27.4)	41.0 (33.3)	<0.001
CARDIA Scale, sedentary hours/week, mean (SD)	56.3 (22.6)	63.2 (23.0)	58.4 (22.6)	54.6 (21.8)	49.6 (20.7)	<0.001
Falls in past 12 months, n (%)						
None	4256 (69.5)	1010 (23.7)	1065 (25.0)	1068 (25.1)	1113 (26.2)	<0.001
One time	1195 (19.5)	298 (24.9)	307 (25.7)	307 (25.7)	283 (23.7)	
Two or more times	675 (11.0)	223 (33.0)	160 (23.7)	157 (23.3)	135 (20.0)	
Falls Efficacy Score, mean (SD)	10.7 (4.1)	12.6 (4.9)	10.6 (3.8)	10.2 (3.7)	9.4 (3.3)	<0.001
Short Physical Performance Battery score, mean (SD)	8.2 (2.5)	6.8 (2.7)	8.1 (2.4)	8.7 (2.2)	9.3 (2.1)	<0.001
Systolic Blood Pressure, mean (SD)	125.7 (14.3)	128.1 (15.2)	126.4 (14.5)	125.2 (14.0)	123.2 (13.2)	<0.001
Diastolic Blood Pressure, mean (SD)	72.5 (8.8)	73.1 (9.5)	72.6 (8.9)	72.3 (8.6)	72.1 (8.0)	0.03
Waist, inches, mean (SD)	35.4 (5.5)	37.4 (5.9)	36.0 (5.2)	34.9 (5.2)	33.3 (4.6)	<0.001
Weight, lbs, mean (SD)	157.9 (34.1)	166.9 (38.2)	160.3 (34.8)	155.9 (31.6)	148.8 (28.5)	<0.001
Body Mass Index, kg/m^2^, mean (SD)	28.1 (5.7)	29.7 (6.3)	28.7 (5.7)	27.7 (5.5)	26.4 (4.8)	<0.001
Body Mass Index Categories, n (%)						
< 25 kg/m^2^	1868 (32.5)	328 (17.6)	400 (21.4)	495 (26.5)	645 (34.5)	<0.001
25–<30 kg/m^2^	2076 (36.2)	474 (22.8)	524 (25.2)	562 (27.1)	516 (24.9)	
≥ 30 kg/m^2^	1796 (31.3)	597 (33.2)	516 (28.7)	395 (22.0)	288 (16.0)	
Medical History						
Stroke	427 (7.0)	164 (38.4)	123 (28.8)	79 (18.5)	61 (14.3)	<0.001
Congestive Heart Failure	124 (2.0)	67 (54.0)	26 (21.0)	22 (17.7)	9 (7.3)	<0.001
Total CVD	1234 (20.1)	441 (35.7)	329 (26.7)	273 (22.1)	191 (15.5)	<0.001
Hip fracture	177 (2.9)	72 (40.7)	49 (27.7)	39 (22.0)	17 (9.6)	<0.001
Any clinical fracture	2060 (33.6)	580 (28.2)	508 (24.7)	508 (24.7)	464 (22.5)	<0.001
Diabetes	1928 (31.5)	603 (31.3)	529 (27.4)	442 (22.9)	354 (18.4)	<0.001
Osteoarthritis	3748 (61.2)	974 (26.0)	960 (25.6)	930 (24.8)	884 (23.6)	0.005
Invasive cancer	942 (15.4)	292 (31.0)	252 (26.8)	220 (23.4)	178 (18.9)	<0.001
Breast cancer	457 (7.5)	138 (30.2)	124 (27.1)	99 (21.7)	96 (21.0)	0.009

*Abbreviations*: *CARDIA* Coronary Artery Risk Development in Young Adults, *CHAMPS* Community Health Activities Model Program for Seniors, *CVD* Cardiovascular disease, *MET* Metabolic equivalents, *OPACH* Objective Physical Activity and Cardiovascular Health, *SD* Standard deviation

## Discussion

The OPACH Study is one of the first, large prospective studies in older women to measure PA objectively using a state-of-the-science triaxial accelerometer. The cohort is unique in its diversity (2,187 Non-Hispanic Black and 1,097 Hispanic/Latina women) and in the richness of adjudicated CVD, cancer, hip fracture and other outcomes. Moreover, physical function, activities of daily living disability, quality of life, and incident hospitalizations are being measured annually through at least the year 2020.

The OPACH study will address gaps in knowledge about PA, falls, and fall-related injuries. Injuries are the most common adverse event from participation in PA [7]. However, evidence is limited about how overall risk of major injuries requiring medical care depends upon level of PA [29]. Despite an extensive literature on exercise and falls in older adults [30], there are no data quantifying fall and fall-related injury risk in relation to accelerometer-measured PA in older women. RCT data clearly show that exercise programs reduce risk of falls in older adults [7], but it is unknown whether these programs might increase risk of other types of injury, for example, through increased exposure to road traffic as an exercising pedestrian. Observational data in older populations performing their usual activities (as opposed to a supervised intervention) are needed to characterize more completely both the benefits and risks of PA, particularly the habitual low levels of PA not typically captured by self-reported questionnaires.

The OPACH Calibration study results clearly showed that traditional cutpoints underestimate the amount of MVPA and overestimate sedentary time in older women [24]. Although we are providing novel information on age and gender appropriate intensity cutpoints for PA, we were unable to design a study that would provide individualized “relative-intensity” cutpoints that account for individual cardiorespiratory fitness. This is an important direction for future research. Our capture of raw accelerometer data in this large population is leading to novel uses of that go beyond defining intensity levels to capture types of PA (walking, sit-to-stand transitions, standing time) using machine-learning algorithms [31], latent class patterns of PA and sedentary behavior, and other inventive summaries. We strongly recommend the capture and storage of raw accelerometer data in all future studies so that new strategies for analyzing the data can be further developed and employed.

The OPACH Study is a cost-effective approach to creating an unparalleled dataset for understanding the health benefits of PA for older women. Our primary objectives focus first on CVD health and falls in older women. However, the addition of accelerometer data to the WHI Program will have tremendous value in studying other phenotypes related to healthy aging, including inflammatory biomarkers, breast and colon cancer, diabetes, and physical disability.

## Additional files

Additional file 1: OPACH PA Questionnaire. (DOCX 351 kb)
