# Supplementary material for: The Objective Physical Activity and Cardiovascular Disease Health in Older Women (OPACH) Study
Source: BMC Public Health. 2017 Feb 14;17:192. doi: 10.1186/s12889-017-4065-6 (PMC5307783; doi:10.1186/s12889-017-4065-6)
Supplement: Additional file 1: — OPACH PA Questionnaire. (DOCX 351 kb) [file 12889_2017_4065_MOESM1_ESM.docx]

| Date Received: -- (MM/DD/YY) | | **- Affix label here-** |
| --- | --- | --- |
| Reviewed By: | | Participant ID: ___ ___ - ___ ___ ___ ___ ___ - ___  First Name ________________________M.I.______  Last Name _________________________________ |
| Contact Type: ¨1 Phone | Visit Type: ¨3 Annual # | |
| ¨2 Mail | ¨4 Non-Routine | |
| ¨3 Visit |  | |
| ¨8 Other |  | |
| **OFFICE USE ONLY** | | |

**These questions ask about physical activity and other habits that may affect your health. Please answer each question as accurately as possible. There are no right or wrong answers.**

**The first questions are about your usual physical activity and exercise. This includes walking and sports, household chores, and lawn work and gardening.**

1. Think about the walking you do outside the home. How often do you walk outside the home
for more than 10 minutes without stopping? **(Mark only one)**

¨0 Rarely or never

¨1 1 to 3 times each month

¨2 1 time each week

¨3 2 to 3 times each week

¨4 4 to 6 times each week

¨5 7 or more times each week

| When you walk outside the home for more than 10 minutes without stopping,  1.1 For how many minutes do you usually walk?  ¨1 Less than 20 minutes  ¨2 20 to 39 minutes  ¨3 40 to 59 minutes  ¨4 1 hour or more |
| --- |
| 1.2 What is your usual speed?  ¨2 Casual strolling or walking (less than 2 miles an hour)  ¨3 Average or normal (2-3 miles an hour)  ¨4 Fairly fast (3-4 miles an hour)  ¨5 Very fast (more than 4 miles an hour)  ¨9 Don’t know  **Go to the next page.** |

2. Not including walking outside the home, how often each week (7 days) do you usually do the exercises listed below?

2.1 STRENUOUS OR VERY HARD EXERCISE (You work up a sweat and your heart beats fast.) For example, aerobics, aerobic dancing, jogging, tennis, swimming laps.

¨0 Rarely or never

| 2.2 How long do you usually exercise like this at one time?  ¨1 Less than 20 minutes  ¨2 20 to 39 minutes  ¨3 40 to 59 minutes  ¨4 1 hour or more |
| --- |

¨1 1 day per week

¨2 2 days per week

¨3 3 days per week

¨4 4 days per week

¨5 5 or more days per week

2.3 MODERATE EXERCISE (Not exhausting). For example, biking outdoors, using an exercise machine (like a stationary bike or treadmill), calisthenics, easy swimming, popular and folk dancing.

¨0 Rarely or never

| 2.4 How long do you usually exercise like this at one time?  ¨1 Less than 20 minutes  ¨2 20 to 39 minutes  ¨3 40 to 59 minutes  ¨4 1 hour or more |
| --- |

¨1 1 day per week

¨2 2 days per week

¨3 3 days per week

¨4 4 days per week

¨5 5 or more days per week

2.5 MILD EXERCISE. For example, slow dancing, bowling or golf.

¨0 Rarely or never

2.6 How long do you usually exercise like this at one time?

¨1 Less than 20 minutes

¨2 20 to 39 minutes

¨3 40 to 59 minutes

¨4 1 hour or more

¨1 1 day per week

¨2 2 days per week

¨3 3 days per week

¨4 4 days per week

¨5 5 or more days per week

3. About how many hours each week do you usually spend doing heavy (strenuous) indoor household chores such as scrubbing floors, sweeping, or vacuuming?

| Less than  1 hour | 1-3 hours | 4-6 hours | 7-9 hours | 10 or more hours |
| --- | --- | --- | --- | --- |
| ¨1 | ¨2 | ¨3 | ¨4 | ¨5 |

4. About how many months during the year do you usually do things in the yard, such as mowing, raking, gardening, or shoveling snow?

|  | Less than  1 month | | | | | 1-3  months | | | | 4-6  months | | | | 7-9  months | | | | 10 or more months | | |  | | | |
| --- | --- | --- | --- | --- | --- | --- | --- | --- | --- | --- | --- | --- | --- | --- | --- | --- | --- | --- | --- | --- | --- | --- | --- | --- |
|  | | ¨1 | | | | | ¨2 | | | | ¨3 | | | | ¨4 | | | | ¨5 | | |  | | |
|  | | |  | | | | |  | | | |  | | | | |  | | | | | |  | |
|  | | |  | 4.1. When you do these things in the yard, how many hours each week do you do them? | | | | | | | | | | | | | | | | | | | |  |
|  | | |  |  | Less than 1 hour | | | | 1-3 hours | | | | 4-6 hours | | | 7-9 hours | | | | 10 or more hours | | | |  |
|  | | |  |  | ¨1 | | | | ¨2 | | | | ¨3 | | | ¨4 | | | | ¨5 | | | |  |
|  | | | |  | | | | | | | | | | | | | | | | | | | | |

5. When you exercise or walk in your usual fashion how would you rate your level of exertion (degree of effort)? Please circle one number.

0 0.5 1 2 3 4 5 6 7 8 9 10 *Maximal*

|  |  |  |  |  |  |  |  |  |  | ● |
| --- | --- | --- | --- | --- | --- | --- | --- | --- | --- | --- |
|  |  |  |  |  |  |  |  |  |  |  |

*Nothing Very Weak Moderate Somewhat Strong Very Very, very*

*at all weak strong (heavy) strong strong*

*(almost*

*Very, very maximal)*

*weak (just noticeable)*

6. Are you able to walk at a normal pace for a half hour (30 minutes) or more?

¨0 No

¨1 Yes

7. Are you able to walk slowly for a half hour (30 minutes) or more?

¨0 No

¨1 Yes

1. During a usual day and night, about how many hours do you spend sitting? Be sure to include the time you spend sitting at work, sitting at the table eating, driving or riding in a car or bus, and sitting up watching TV or talking.

| Less than 4 hours | 4-5 hours | 6-7 hours | 8-9 hours | 10-11 hours | 12-13 hours | 14-15 hours | 16 or more hours |
| --- | --- | --- | --- | --- | --- | --- | --- |
| ¨1 | ¨2 | ¨3 | ¨4 | ¨5 | ¨6 | ¨7 | ¨8 |

9. During a usual day and night, about how many hours do you spend sleeping or lying down with your feet up? Be sure to include the time you spend sleeping or trying to sleep at night, resting or napping, and lying down watching TV.

| Less than 4 hours | 4-5 hours | 6-7 hours | 8-9 hours | 10-11 hours | 12-13 hours | 14-15 hours | 16 or more hours |
| --- | --- | --- | --- | --- | --- | --- | --- |
| ¨1 | ¨2 | ¨3 | ¨4 | ¨5 | ¨6 | ¨7 | ¨8 |

*10 About how many hours of sleep did you get on a typical night during the past 4 weeks?*

| 5 hours or less | 6 hours | 7 hours | 8 hours | 9 hours | 10 or more  Hours |  |  |
| --- | --- | --- | --- | --- | --- | --- | --- |
| ¨1 | ¨2 | ¨3 | ¨4 | ¨5 | ¨6 |  |  |

11. On a typical **WEEKDAY**, how much time do you spend (from when you wake up until you go to bed) doing the following? Please check one answer per question.

|  | **None** | **15 min. or less** | **30 min.** | **1**  **hours** | **2**  **hours** | **3**  **hours** | **4**  **hours** | **5**  **hours** | **6 hrs. or more** |
| --- | --- | --- | --- | --- | --- | --- | --- | --- | --- |
| 11.1 Sitting while watching television (including videos on VCR/DVD). | ¨1 | ¨2 | ¨3 | ¨4 | ¨5 | ¨6 | ¨7 | ¨10 | ¨11 |
| 11.2 Sitting while using the computer for non-work activities or playing video games. | ¨1 | ¨2 | ¨3 | ¨4 | ¨5 | ¨6 | ¨7 | ¨10 | ¨11 |
| 11.3 Sitting while doing non-computer office work or paperwork not related to your job (paying bills, etc.). | ¨1 | ¨2 | ¨3 | ¨4 | ¨5 | ¨6 | ¨7 | ¨10 | ¨11 |
| 11.4 Sitting listening to music, reading a book or magazine, or doing arts and crafts. | ¨1 | ¨2 | ¨3 | ¨4 | ¨5 | ¨6 | ¨7 | ¨10 | ¨11 |
| 11.5 Sitting and talking on the phone or texting. | ¨1 | ¨2 | ¨3 | ¨4 | ¨5 | ¨6 | ¨7 | ¨10 | ¨11 |
| 11.6 Sitting in a car, bus, train, or other mode of transportation | ¨1 | ¨2 | ¨3 | ¨4 | ¨5 | ¨6 | ¨7 | ¨10 | ¨11 |

12. On a typical **WEEKEND DAY**, how much time do you spend (from when you wake up until you go to bed) doing the following? Please check one answer per question.

|  | **None** | **15 min. or less** | **30 min.** | **1**  **hours** | **2**  **hours** | **3**  **hours** | **4**  **hours** | **5**  **hours** | **6 hrs. or more** |
| --- | --- | --- | --- | --- | --- | --- | --- | --- | --- |
| 12.1 Sitting while watching television (including videos on VCR/DVD). | ¨1 | ¨2 | ¨3 | ¨4 | ¨5 | ¨6 | ¨7 | ¨10 | ¨11 |
| 12.2 Sitting while using the computer for non-work activities or playing video games. | ¨1 | ¨2 | ¨3 | ¨4 | ¨5 | ¨6 | ¨7 | ¨10 | ¨11 |
| 12.3 Sitting while doing non-computer office work or paperwork not related to your job (paying bills, etc.). | ¨1 | ¨2 | ¨3 | ¨4 | ¨5 | ¨6 | ¨7 | ¨10 | ¨11 |
| 12.4 Sitting listening to music, reading a book or magazine, or doing arts and crafts. | ¨1 | ¨2 | ¨3 | ¨4 | ¨5 | ¨6 | ¨7 | ¨10 | ¨11 |
| 12.5 Sitting and talking on the phone or texting. | ¨1 | ¨2 | ¨3 | ¨4 | ¨5 | ¨6 | ¨7 | ¨10 | ¨11 |
| 12.6 Sitting in a car, bus, train, or other mode of transportation | ¨1 | ¨2 | ¨3 | ¨4 | ¨5 | ¨6 | ¨7 | ¨10 | ¨11 |

13. **These questions ask about how concerned you are about the possibility of falling. When you usually do each of these activities, how concerned are you that you might fall?** (If you currently don’t do the activity, think about how concerned about falling you would be IF you did the activity.)

|  | **Not at all concerned** | **Somewhat concerned** | **Fairly concerned** | **Very concerned** |
| --- | --- | --- | --- | --- |
| 13.1 Getting dressed or undressed | ¨1 | ¨2 | ¨3 | ¨4 |
| 13.2 Taking a bath or shower | ¨1 | ¨2 | ¨3 | ¨4 |
| 13.3 Getting in or out of a chair | ¨1 | ¨2 | ¨3 | ¨4 |
| 13.4 Going up or down stairs | ¨1 | ¨2 | ¨3 | ¨4 |
| 13.5 Reaching for something above your head or on the ground | ¨1 | ¨2 | ¨3 | ¨4 |
| 13.6 Walking up or down a slope | ¨1 | ¨2 | ¨3 | ¨4 |
| 13.7 Going out to a social event (e.g., religious service, family gathering, or club meeting) | ¨1 | ¨2 | ¨3 | ¨4 |

14. If you fell when moving around inside your home, how confident are you that someone would be able to quickly help you?

¨1 Not at all confident

¨2 Somewhat confident

¨3 Very confident

¨9 Don’t Know/Not Sure

15. If you fell when you are away from your home, how confident are you that someone would be able to quickly help you?

¨1 Not at all confident

¨2 Somewhat confident

¨3 Very confident

¨9 Don’t Know/Not Sure

16. Do you ever use a cane, walker, or similar device to assist you when you are walking?

¨1 Never

¨2 Occasionally

¨3 Frequently or all the time

17. Does the place where you live require you to climb stairs, either inside or outside the home?

¨0 No

¨1 Yes, there are 1 to 4 stairs

17.1 Do all or most of these stairs have handrails?

¨0 No

¨1 Yes

¨2 Yes, there are 5 or more stairs

18. Are you currently doing, or have you ever done, a falls prevention program?

¨0 No

18.1 What did the program provide? **(Mark all that apply.)**

¨1 Exercises, such as physical therapy

¨2 Assessment of fall hazards in the home

¨3 Written materials and/or discussion about preventing falls

¨4 Review of medications

¨5 Use of devices (better footware, hip protectors, cane, etc.)

¨6 Other:

¨1 Yes

**Go to the next page.**

19. Have you made modifications to your home to reduce your risk of falling?

¨0 No

19.1 What have you done? **(Mark all that apply.)**

¨1 Grab bars or handrails anywhere in the home

¨2 Bath mat or nonskid strips in bathroom areas

¨3 Better lighting

¨4 Removed rugs or taped them down to the floor

¨5 Other: _________________________________________

¨1 Yes

20. Have you had a fall in the **past 12 months**? By a “fall”, we mean

- Fell all the way to the floor or the ground, or
- Fell and hit an object like a chair or stair

¨0 No **Go to Question 27.**

¨1 Yes

21. How many times have you fallen in the **past 12 months**? (If you are unsure, make your best guess.):

¨1 One time

¨2 Two or three times

¨3 Four or five times

¨4 Six or more times

| 22. **At the time of your most recent fall, were you:** | **Yes** | **No** |
| --- | --- | --- |
| 22.1 Walking outside the home? | ¨1 | ¨0 |
| 22.2 Doing strenuous exercise (but not walking outside the home)? Strenuous meanse you work up a sweat and would be exhausted by prolonged participation. For example, aerobics, aerobic dancing, jogging, tennis, swimming laps. | ¨1 | ¨0 |
| 22.3 Doing moderate exercise (but not walking outside the home)? Moderate means exercise that is not exhausting. For example, biking outdoors, using an exercise machine (like a stationary bike or treadmill), calisthenics, easy swimming, popular and folk dancing. | ¨1 | ¨0 |
| 22.4 Doing mild exercise? For example, slow dancing, bowling, or golf. | ¨1 | ¨0 |
| 22.5 Doing other exercise (not previously listed)? | ¨1 | ¨0 |
| 22.6 Doing strenuous indoor household chores (such as scrubbing floors, sweeping, or vacuuming)? | ¨1 | ¨0 |
| 22.7 Working in the yard (such as mowing, raking, gardening, or shoveling snow)? | ¨1 | ¨0 |

23. Were you injured as a result of a fall in the **past 12 months**?

¨0 No **Go to Question 27.**

¨1 Yes

23.1 Please indicate what types of injuries. **(Mark all that apply.)**

¨1 Fracture

¨2 Laceration/Cut

¨3 Bruising

¨4 Sprained or strained joint (wrist, knee, ankle, etc.)

¨5 Other injury (Please specify: ______________________)

23.2 Did you injure your head?

¨0 No

¨1 Yes

24. Did you receive treatment from a doctor for an injury from a fall in the **past 12 months**?

¨0 No

¨1 Yes

24.1 Did you stay in a hospital overnight for treatment of an injury from a fall?

¨0 No

¨1 Yes

25. Did you limit your usual activities for more than a day because of an injury from a fall in the
**past 12 months**?

¨0 No

¨1 Yes

| 26. **When you were injured from falling in the past 12 months,  were you:** | **Yes** | **No** |
| --- | --- | --- |
| 26.1 Walking outside the home? | ¨1 | ¨0 |
| 26.2 Doing strenuous exercise (but not walking outside the home)? Strenuous meanse you work up a sweat and would be exhausted by prolonged participation. For example, aerobics, aerobic dancing, jogging, tennis, swimming laps. | ¨1 | ¨0 |
| 26.3 Doing moderate exercise (but not walking outside the home)? Moderate means exercise that is not exhausting. For example, biking outdoors, using an exercise machine (like a stationary bike or treadmill), calisthenics, easy swimming, popular and folk dancing. | ¨1 | ¨0 |
| 26.4 Doing mild exercise? For example, slow dancing, bowling, or golf. | ¨1 | ¨0 |
| 26.5 Doing other exercise (not previously listed)? | ¨1 | ¨0 |
| 26.6 Doing strenuous indoor household chores (such as scrubbing floors, sweeping, or vacuuming)? | ¨1 | ¨0 |
| 26.7 Working in the yard (such as mowing, raking, gardening, or shoveling snow)? | ¨1 | ¨0 |

27. When was the house/structure you live in originally built?

¨1 Before 1946

¨2 1946-1973

¨3 1974 to 1994

¨4 1995 to present

¨9 Don’t know

28. Is your neighborhood primarily:

¨1 Residential

¨2 Commercial or a mix of residential and commercial

¨9 Don’t know

| 29. **How much do you agree or disagree with these statements:** | **Strongly**  **Disagree** | **Somewhat Disagree** | **Somewhat Agree** | **Strongly**  **Agree** |
| --- | --- | --- | --- | --- |
| 29.1 There is a high crime rate in my neighborhood. | ¨1 | ¨2 | ¨3 | ¨4 |
| 29.2 The crime rate in my neighborhood makes it unsafe to go on walks **during the day**. | ¨1 | ¨2 | ¨3 | ¨4 |
| 29.3 The crime rate in my neighborhood makes it unsafe to go on walks **at night**. | ¨1 | ¨2 | ¨3 | ¨4 |

**This set of questions are to help us understand the full range of activites you are doing. These questions are about activities that you may have done in the past 4 weeks. The questions on the following pages are similar to the example shown below.**

## If you DID NOT do the activity:

- Check the **NO** box and move to the next question

## If you DID the activity in the past 4 weeks:

Step #1 Check the **YES** box.

Step #2 Think about how many TIMES a week you usually did it, and write your response in the space provided.

Step #3 Circle how many TOTAL HOURS in a typical week you did the activity.

**Here is an example** of how Mrs. Jones would answer the first question: Mrs. Jones usually visits her friends Maria and Olga twice a week. She usually spends one hour on Monday with Maria and two hours on Wednesday with Olga. Therefore, the total hours a week that she visits with friends is 3 hours a week.

| **In a typical week during the past 4 weeks, did you . . .** | **No** | **Yes** | | **How many TIMES**  **a week?** |  | **How many TOTAL hours a week did you usually do it?** | | | | | |
| --- | --- | --- | --- | --- | --- | --- | --- | --- | --- | --- | --- |
|  |  |  |  |  |  | **Less than 1 hour** | **1-2.5**  **hours** | **3-4.5 hours** | **5-6.5 hours** | **7-8.5 hours** | **9 or more hours** |
| Visit with friends or family (other than those you live with)? | ¨0 | ⌧1 |  | **0 2** |  | ¨1 | ¨2 | ⌧3 | ¨4 | ¨5 | ¨6 |

| 30. **In a typical week during the past 4 weeks, did you . . .** | **No** | | **Yes** | | | **How many TIMES**  **a week?** | |  | | **How many TOTAL hours a week did you usually do it?** | | | | | | | | | | | |  |
| --- | --- | --- | --- | --- | --- | --- | --- | --- | --- | --- | --- | --- | --- | --- | --- | --- | --- | --- | --- | --- | --- | --- |
|  |  |  |  |  |  |  |  |  |  | **Less than 1 hour** | | **1-2.5 hours** | | **3-4.5 hours** | | **5-6.5 hours** | | **7-8.5 hours** | | **9 or more hours** | |  |
| 30.1 Visit with friends or family (other than those you live with)? | | ¨0 | | ¨1 |  | |  | |  | | ¨1 | | ¨2 | | ¨3 | | ¨4 | | ¨5 | | ¨6 | |
| - 1. Go to the senior center? | | ¨0 | | ¨1 |  | |  | |  | | ¨1 | | ¨2 | | ¨3 | | ¨4 | | ¨5 | | ¨6 | |
| - 1. Do volunteer work? | | ¨0 | | ¨1 |  | |  | |  | | ¨1 | | ¨2 | | ¨3 | | ¨4 | | ¨5 | | ¨6 | |
| - 1. Attend church or take part in church activities? | | ¨0 | | ¨1 |  | |  | |  | | ¨1 | | ¨2 | | ¨3 | | ¨4 | | ¨5 | | ¨6 | |
| - 1. Attend other club or group meetings? | | ¨0 | | ¨1 |  | |  | |  | | ¨1 | | ¨2 | | ¨3 | | ¨4 | | ¨5 | | ¨6 | |

| **In a typical week during the past 4 weeks, did you . . .** | **No** | **Yes** | | **How many TIMES**  **a week?** |  | **How many TOTAL hours a week did you usually do it?** | | | | | |
| --- | --- | --- | --- | --- | --- | --- | --- | --- | --- | --- | --- |
|  |  |  |  |  |  | **Less than 1 hour** | **1 to 2.5 hours** | **3 to 4.5 hours** | **5 to 6.5 hours** | **7 to 8.5**  **hours** | **9 or more hours** |
| 30.6 Use a computer? | ¨0 | ¨1 |  |  |  | ¨1 | ¨2 | ¨3 | ¨4 | ¨5 | ¨6 |
| 30.7 Dance (such as square, folk, line, ballroom) (do not count aerobic dance here)? | ¨0 | ¨1 |  |  |  | ¨1 | ¨2 | ¨3 | ¨4 | ¨5 | ¨6 |
| 30.8 Do woodworking, needlework, drawing, or other arts or crafts? | ¨0 | ¨1 |  |  |  | ¨1 | ¨2 | ¨3 | ¨4 | ¨5 | ¨6 |
| 30.9 Play golf, carrying or pulling your equipment (count walking time only)? | ¨0 | ¨1 |  |  |  | ¨1 | ¨2 | ¨3 | ¨4 | ¨5 | ¨6 |
| 30.10 Play golf, riding a cart (count walking time only)? | ¨0 | ¨1 |  |  |  | ¨1 | ¨2 | ¨3 | ¨4 | ¨5 | ¨6 |
| 30.11 Attend a concert, movie, lecture, or sport event? | ¨0 | ¨1 |  |  |  | ¨1 | ¨2 | ¨3 | ¨4 | ¨5 | ¨6 |
| 30.12 Play cards, bingo, or board games with other people? | ¨0 | ¨1 |  |  |  | ¨1 | ¨2 | ¨3 | ¨4 | ¨5 | ¨6 |
| 30.13 Shoot pool or billiards? | ¨0 | ¨1 |  |  |  | ¨1 | ¨2 | ¨3 | ¨4 | ¨5 | ¨6 |
| 30.14 Play singles tennis (do not count doubles)? | ¨0 | ¨1 |  |  |  | ¨1 | ¨2 | ¨3 | ¨4 | ¨5 | ¨6 |
| 30.15 Play doubles tennis (do not count singles)? | ¨0 | ¨1 |  |  |  | ¨1 | ¨2 | ¨3 | ¨4 | ¨5 | ¨6 |
| 30.16 Skate (ice, roller, in-line)? | ¨0 | ¨1 |  |  |  | ¨1 | ¨2 | ¨3 | ¨4 | ¨5 | ¨6 |
| 30.17 Play a musical instrument? | ¨0 | ¨1 |  |  |  | ¨1 | ¨2 | ¨3 | ¨4 | ¨5 | ¨6 |
| 30.18 Read? | ¨0 | ¨1 |  |  |  | ¨1 | ¨2 | ¨3 | ¨4 | ¨5 | ¨6 |
| 30.19 Do heavy work around the house (such as washing windows, cleaning gutters)? | ¨0 | ¨1 |  |  |  | ¨1 | ¨2 | ¨3 | ¨4 | ¨5 | ¨6 |

| **In a typical week during the past 4 weeks, did you . . .** | **No** | **Yes** | | **How many TIMES**  **a week?** |  | **How many TOTAL hours a week did you usually do it?** | | | | | | |
| --- | --- | --- | --- | --- | --- | --- | --- | --- | --- | --- | --- | --- |
|  |  |  |  |  |  | **Less than 1 hour** | **1 to 2.5 hours** | **3 to 4.5 hours** | | **5 to 6.5 hours** | **7 to 8.5 hours** | **9 or more hours** |
| 30.20 Do light work around the house (such as sweeping or vacuuming)? | ¨0 | ¨1 |  |  |  | ¨1 | ¨2 | ¨3 | | ¨4 | ¨5 | ¨6 |
| 30.21 Do heavy gardening (such as spading, raking)? | ¨0 | ¨1 |  |  |  | ¨1 | ¨2 | ¨3 | | ¨4 | ¨5 | ¨6 |
| 30.22 Do light gardening (such as watering plants)? | ¨0 | ¨1 |  |  |  | ¨1 | ¨2 | ¨3 | | ¨4 | ¨5 | ¨6 |
| 30.23 Work on your car, truck, lawn mower, or other machinery? | ¨0 | ¨1 |  |  |  | ¨1 | ¨2 | ¨3 | | ¨4 | ¨5 | ¨6 |
| 30.24 Jog or run (including use of treadmill)? | ¨0 | ¨1 |  |  |  | ¨1 | ¨2 | ¨3 | | ¨4 | ¨5 | ¨6 |
| 30.25 Walk uphill or hike uphill (count only uphill part; include use of treadmill)? | ¨0 | ¨1 |  |  |  | ¨1 | ¨2 | ¨3 | | ¨4 | ¨5 | ¨6 |
| 30.26 Walk fast or briskly for exercise (do not count walking leisurely or uphill; include use of treadmill)? | ¨0 | ¨1 |  |  |  | ¨1 | ¨2 | ¨3 | | ¨4 | ¨5 | ¨6 |
| 30.27 Walk to do errands (such as to/from a store or to take children to school (count walk time only)? | ¨0 | ¨1 |  |  |  | ¨1 | ¨2 | ¨3 | | ¨4 | ¨5 | ¨6 |
| 30.28 Walk leisurely for exercise or pleasure? | ¨0 | ¨1 |  |  |  | ¨1 | ¨2 | ¨3 | | ¨4 | ¨5 | ¨6 |
| 30.29 Ride a bicycle or stationary cycle? | ¨0 | ¨1 |  |  |  | ¨1 | ¨2 | ¨3 | | ¨4 | ¨5 | ¨6 |
| 30.30 Do other aerobic machines such as rowing, or step machines (do not count treadmill or stationary cycle)? | ¨0 | ¨1 |  |  |  | ¨1 | ¨2 | ¨3 | | ¨4 | ¨5 | ¨6 |
| **In a typical week during the past 4 weeks, did you . . .** | **No** | **Yes** | | **How many TIMES**  **a week?** |  | **How many TOTAL hours a week did you usually do it?** | | | | | | |
|  |  |  |  |  |  | **Less than 1 hour** | **1 to 2.5 hours** | | **3 to 4.5 hours** | **5 to 6.5 hours** | **7 to 8.5 hours** | **9 or more hours** |
| 30.31 Do water exercises (do not count other swimming)? | ¨0 | ¨1 |  |  |  | ¨1 | ¨2 | ¨3 | | ¨4 | ¨5 | ¨6 |
| 30.32 Swim moderately or fast? | ¨0 | ¨1 |  |  |  | ¨1 | ¨2 | ¨3 | | ¨4 | ¨5 | ¨6 |
| 30.33 Swim gently? | ¨0 | ¨1 |  |  |  | ¨1 | ¨2 | ¨3 | | ¨4 | ¨5 | ¨6 |
| 30.34 Do stretching or flexibility exercises (do not count yoga or Tai-chi)? | ¨0 | ¨1 |  |  |  | ¨1 | ¨2 | ¨3 | | ¨4 | ¨5 | ¨6 |
| 30.35 Do yoga or Tai-chi? | ¨0 | ¨1 |  |  |  | ¨1 | ¨2 | ¨3 | | ¨4 | ¨5 | ¨6 |
| 30.36 Do aerobics or aerobic dancing? | ¨0 | ¨1 |  |  |  | ¨1 | ¨2 | ¨3 | | ¨4 | ¨5 | ¨6 |
| 30.37 Do moderate to heavy strength training (such as hand-held weights of more than 5 lbs., weight machines, or push-ups)? | ¨0 | ¨1 |  |  |  | ¨1 | ¨2 | ¨3 | | ¨4 | ¨5 | ¨6 |
| 30.38 Do light strength training (such as hand-held weights of 5 lbs. or less or elastic bands)? | ¨0 | ¨1 |  |  |  | ¨1 | ¨2 | ¨3 | | ¨4 | ¨5 | ¨6 |
| 30.39 Do general conditioning exercises, such as light calisthenics or chair exercises (do not count strength training)? | ¨0 | ¨1 |  |  |  | ¨1 | ¨2 | ¨3 | | ¨4 | ¨5 | ¨6 |
| 30.40 Play basketball, soccer, or racquetball (do not count time on sidelines)? | ¨0 | ¨1 |  |  |  | ¨1 | ¨2 | ¨3 | | ¨4 | ¨5 | ¨6 |
| 30.41 Do other types of physical activity not previously mentioned (please specify)? | ¨0 | ¨1 |  |  |  | ¨1 | ¨2 | ¨3 | | ¨4 | ¨5 | ¨6 |

31. What is the date you finished this form?

--

month day year

| **OFFICE USE ONLY** |
| --- |
| Form Administration |
| ¨1 Self |
| ¨2 Group |
| ¨3 Interview |
| ¨4 Assistance |
